# Immunization Strategies Targeting Newly Arrived Migrants in Non-EU Countries of the Mediterranean Basin and Black Sea

**DOI:** 10.3390/ijerph14050459

**Published:** 2017-04-25

**Authors:** Cristina Giambi, Martina Del Manso, Maria Grazia Dente, Christian Napoli, Carmen Montaño-Remacha, Flavia Riccardo, Silvia Declich, Network for the control of cross-border health threats in the Mediterranean Basin and Black Sea for the ProVacMed project

**Affiliations:** 1 National Center for Epidemiology, Surveillance and Health Promotion, National Institute of Health (Istituto Superiore di Sanità, ISS), viale Regina Elena, 299, 00161 Rome, Italy; martina.delmanso@iss.it (M.D.M.); mariagrazia.dente@iss.it (M.G.D.); flavia.riccardo@iss.it (F.R.); silvia.declich@iss.it (S.D.); 2 Department of Medical Surgical Sciences and Traslational Medicine, “Sapienza” University of Rome, Via di Grottarossa, 1035/1039, 00189 Rome, Italy; christian.napoli@uniroma1.it; 3 Department of Epidemiology, Andalusian Regional Ministry of Health, Avenida de la innovaciòn s/n, 41020 Sevilla, Spain; mariac.montano.sspa@juntadeandalucia.es; 4 Network for the Control of Cross-Border Health Threats in the Mediterranean Basin and Black Sea for the ProVacMed Project—Coordination Centre, National Institute of Health (Istituto Superiore di Sanità, ISS), viale Regina Elena 299, 00161 Rome, Italy; episouth@iss.it

**Keywords:** migrants, vaccination, infectious diseases, Mediterranean Basin and Black Sea

## Abstract

*Background*: The World Health Organization recommends that host countries ensure appropriate vaccinations to refugees, asylum seekers and migrants. However, information on vaccination strategies targeting migrants in host countries is limited. *Methods*: In 2015–2016 we carried out a survey among national experts from governmental bodies of 15 non-EU countries of the Mediterranean and Black Sea in order to document and share national vaccination strategies targeting newly arrived migrants. *Results*: Four countries reported having regulations/procedures supporting the immunization of migrants at national level, one at sub-national level and three only targeting specific population groups. Eight countries offer migrant children all the vaccinations included in their national immunization schedule; three provide only selected vaccinations, mainly measles and polio vaccines. Ten and eight countries also offer selected vaccinations to adolescents and adults respectively. Eight countries provide vaccinations at the community level; seven give priority vaccines in holding centres or at entry sites. Data on administered vaccines are recorded in immunization registries in nine countries. *Conclusions*: Although differing among countries, indications for immunizing migrants are in place in most of them. However, we cannot infer from our findings whether those strategies are currently functioning and whether barriers to their implementation are being faced. Further studies focusing on these aspects are needed to develop concrete and targeted recommendations for action. Since migrants are moving across countries, development of on-line registries and cooperation between countries could allow keeping track of administered vaccines in order to appropriately plan immunization series and avoid unnecessary vaccinations.

## 1. Introduction

The World Health Organization (WHO) states that there is no systematic association between migration and the importation of infectious diseases. However, conditions that migrants face during their exhausting journeys, such as lack of sufficient water, and inadequate shelter and sanitation, increase the risk of acquiring communicable diseases [1]. In addition, crowded settings such as holding centres face an increased risk of outbreaks due to diseases such as measles, influenza, varicella and meningococcal disease. This risk depends on the length of stay and on concurrent sanitary conditions [2,3,4,5,6].

Unfortunately, despite the widespread availability of vaccines, misconceptions about vaccines and, consequently, vaccination hesitancy are globally increasing in the general population [7,8,9]. Many host countries have sub-optimal vaccination coverage rates with, in some cases, decreasing trends [10]. This is also the case for consolidated immunizations programmes like poliomyelitis vaccination. For example, in Italy vaccination coverage among children has been decreasing since 2012. In 2015, the vaccination coverage rate for poliomyelitis in children at 24 months of age in this country dropped below 95% [11]. This comes at a time when the WHO European Region is at risk for a poliomyelitis outbreak. The Regional Certification Commission for Poliomyelitis Eradication has repeatedly identified Bosnia and Herzegovina, Romania and Ukraine as at high risk for transmission in the event of wild poliovirus importation [12]. An outbreak of circulating vaccine-derived poliovirus occurred in Ukraine in September 2015 [13]. Also, although targeted for elimination, in 2015 WHO counted 30,762 measles cases in the European Region (nearly doubled in comparison to the previous year) [14] and measles outbreaks continue to occur in many countries of the Region independently of refugee and migrant population movement [12,14], confirming that pockets of susceptible individuals are still present in recipients countries.

Decreasing immunization coverage combined with access barriers to immunization in countries of transit and destination could hypothetically lead to a scenario where rapid inflows of large numbers of unvaccinated people could increase existing immunity gaps. It is very hard to infer the occurrence of measles or other vaccine preventable diseases (VPD) among migrants in Europe due to the incompleteness of surveillance in relation to migrant health [6,15]. However, outbreaks of measles and other VPDs have been documented in migrant settings within destination/transit countries of Mediterranean migration routes [1,16,17,18,19]. In European countries, evidence of low seroprevalence rates for several VPDs among high risk migrant groups and refugee children [20,21] and of lower vaccination coverage among regular migrant populations compared with local populations [22] have also been documented. This combined evidence suggests that mobile populations across the Mediterranean migration routes may be vulnerable to VPDs as a result of low vaccination coverage in their countries of origin and/or poor access to immunization in countries of transit/destination.

For this reason, in 2015 WHO-UNHCR-UNICEF jointly stated that refugees and asylum seekers should have non-discriminatory, equitable access to vaccination, irrespective of their legal status and regardless of their country of origin [12].

While population movement across the Mediterranean Basin has historical origins, in the last five years migration flows across the Mediterranean Region have increased [23,24]. In 2016, according to the European Agency for the Management of Operational Cooperation at the External Borders of the Member States of the European Union (Frontex), migration routes across non- European Union (EU) countries of the Mediterranean and Black Sea accounted for 487,717 irregular crossings to the EU (98% of all irregular sea crossings to the EU) [25].

An increasing number of incoming migrants, with different migration statuses and diverse health needs, is challenging public health authorities in countries within the Mediterranean migratory system to ensure equitable access to disease prevention and control services, including for vaccine preventable diseases. This concern, initially mainly of countries of destination, is becoming broader as countries on both sides of the Mediterranean basin, traditionally of transit, are becoming long term or final destinations for a growing number of migrants [26,27]. Formal and/or informal barriers to services could be hindering this access. Evidence from a cross-sectional study conducted in 2009 among 22 countries bordering the Mediterranean (EU and non EU) [28] has shown that, although eligibility of migrants to immunization services offered as part of national immunization programs is almost universal, informal barriers to immunization services (e.g., language, information and cultural barriers) are a widespread issue.

Current recommendations by WHO-UNHCR-UNICEF [12] state that refugees, asylum seekers and migrants should be vaccinated without necessary delay in the European region according to the immunization schedule of the host countries. However, since access to a full immunization schedule through follow up vaccinations is difficult to ensure while people are on the move, measles/mumps/rubella (MMR) and polio vaccines should be prioritized.

This general policy needs implementation strategies adapted at country level to meet the challenges and opportunities of each context. Furthermore, national strategies need to be shaped into procedures that involve deliberate actions to achieve the goals set out in the strategy and into regulations that imply an organization setting out the rules and monitoring their implementation [29,30]. Therefore, existence of regulations, procedures and national strategic and implementation plans indicates that a general policy has been translated at national level.

Information on immunization strategies targeting newly arrived migrants in all countries bordering the Southern Mediterranean and Black Sea is not readily available. Following initial actions carried out during the EpiSouth [31,32] and MedPremier (Preparedness in the Mediterranean Region) [27] projects, in 2015–2016 the “VACcination PROgrammes in the MEDiterranean area” (ProVacMed) Project [33] was implemented to enhance and share the knowledge on the control of VPDs in the Mediterranean Area and Black Sea countries. In this project, existing strategies for immunization of newly arrived migrants in non-EU countries of this Region were documented in order to address this information gap and promote the sharing of information and experiences.

The ProVacMed study [33], as the other projects mentioned above [27,28,31,32], was coordinated by the Italian Public Health Institute (Istituto Superiore di Sanità) that conducted the survey and promoted interactions among the experts of the Network.

## 2. Materials and Methods

The EpiSouth and EpiSouth Plus projects [31,32] established a Network for the control of cross-border health threats in the Mediterranean Basin and Middle East that involved officially appointed experts in communicable diseases (Focal Points) in charge of surveillance and control policies in the Ministries of Health or Public Health Institutions. Seventeen non-EU countries were involved: Albania, Algeria, Bosnia & Herzegovina, Egypt, Israel, Jordan, Kosovo (designation without prejudice to positions on status, in line with UNSC 1244 and the ICJ Opinion on the Kosovo declaration of independence), Lebanon, Libya, Montenegro, Morocco, Palestine, Republic of Macedonia-FYROM, Serbia, Syria, Tunisia and Turkey.

At the end of EpiSouth Plus Project (2014), the Network identified further priorities to address [27,34] and expanded to also include four countries of Black Sea region (Armenia, Georgia, Moldavia and Ukraine). Since then the Network was known as “Network for the control of cross-border health threats in the Mediterranean Basin and Black Sea”.

While working on these new priorities, the need of the “ProVacMed” study was identified and discussed with the Focal Points (FPs) during the Network Meeting on the 28–29 May 2015 [35]. Each country of the Network was invited to participate in the study and each FP was asked to identify, within their institutions, experts directly involved in the monitoring and evaluation of national immunization programs and/or migrant health in their country. Therefore, all appointed contact persons were national experts in the field of vaccination and/or migrant health in the Ministries of Health or Public Health Institutions of their countries.

Between February 2015 and August 2016 we carried out a cross-sectional survey among the defined contact persons for the ProVacMed project. The aim of the study was to explore the existence of national regulations, procedures and implementation strategies supporting immunisation of newly arrived migrants in non-EU countries of the Mediterranean Basin and Middle East and Black Sea. As reported in previous studies [27,36], we defined newly arriving migrants as persons, other than travellers or tourists, who had arrived in the previous year (less than 12 months) to a country other than their usual residence.

To collect this information, we developed a questionnaire (in Excel format) addressing:

(i) if regulations/procedures supporting immunization of newly arrived migrants exist in participating countries (no, yes at national level, yes only in some areas, other);
(ii) whether the countries verify migrants’ immunisation status before offering vaccinations (no, yes, only for certain population groups, other);
(iii) how immunization status is verified (anamnesis evaluation, immunization card verification, laboratory tests only if vaccination status is unknown, laboratory tests independently of vaccination status) for each VPD;
(iv) which vaccinations (all vaccinations included in the NIP or specific vaccines) are offered to certain target age groups, defined as children (≤10 years), adolescents (11–18 years), adults (>18 years), and/or specific population groups (to be described by the focal point as open text);
(v) what are the sites for vaccination delivery to newly arrived migrants: entry level (e.g., borders, points of entry); holding level (e.g., migration centres/camps); community level (vaccination after arrival and partial integration to the community in the receiving country: e.g., in the primary health care centres or vaccination services) for each vaccine;
(vi) whether countries record information on administered vaccines (no, in individual vaccination cards, in electronic/paper registries).

For questions (iii), (iv) and (v) an exhaustive list of vaccines was proposed and respondents were asked to provide the information required for each vaccine.

In December 2015, in order to verify if the questionnaire was clear, understandable and easy to fill, it was piloted in two countries and modified accordingly.

In January 2016, the final version of the questionnaire was electronically sent to the expert contact persons, who coordinated the collection of information for the questionnaire in their country, also involving other national experts when appropriate. Those who did not reply to the questionnaire after the initial contact were reminded by email or by phone. Data collection was completed in May 2016.

We carried out a descriptive analysis of collected information. We performed a frequency analysis for all the categorical variables collected and summarized the proportions of responses.

## 3. Results

Among the 21 non-EU countries involved in the Network for the control of cross-border health threats in the Mediterranean Basin and Black Sea, the following 18 identified an expert working in the vaccination and/or migrant health fields to involve in the ProVacMed study: Albania, Algeria, Armenia, Bosnia & Herzegovina, Egypt, Georgia, Israel, Jordan, Kosovo, Lebanon, Libya, Moldavia, Montenegro, Palestine, Republic of Macedonia-FYROM, Serbia, Tunisia, and Ukraine. All except Lebanon, Libya and Montenegro replied to the survey (15/18, 83%).

### 3.1. Regulations/Procedures Supporting Immunization of Newly Arrived Migrants

Four countries (Albania, Israel, Palestine and Serbia) reported having regulations/procedures supporting immunization of newly arrived migrants at the national level. Additionally, in Israel, provisions for specific population groups are in place: for Ethiopian Jews and undocumented migrants aged 0–14 years coming from the horn of Africa and for undocumented migrants aged 0–17 years held in detention facilities.

Three other countries reported regulations only for specific population groups. Recommendations exist in Georgia for people coming from Nigeria, Pakistan, Afghanistan and Syria, in Jordan for Syrian refugees and in Armenia for identifying undocumented migrants, through door to door visits twice a year (Table 1).

In Algeria, specific regulations/procedures supporting immunization of newly arrived migrants are in place only in the southern border regions, where migration centres are more concentrated. In these centres, migrants systematically receive medical attention, care and immunization.

Six countries (Bosnia and Herzegovina, Egypt, Moldova, Republic of Macedonia-FYROM, Tunisia and Ukraine) reported not having specific regulations/procedures for the immunization of newly arrived migrants. Kosovo stated that, according to the plan prepared by the Ministry of Health in case of a migration influx, immunization priority would be given to the vaccination of children aged less than 5 years against measles and poliomyelitis, in agreement with WHO policy. Egypt, Moldova, the Republic of Macedonia-FYROM and Tunisia, while reporting no specific regulations/procedures targeting newly arrived migrants, provided information on vaccinations offered to this population group, as summarized in Table 2 and Table 3.

### 3.2. Verification of Immunization Status of Newly Arrived Migrants

The following descriptive analysis on the verification of migrants’ immunization status, vaccinations offered to newly arrived migrants, target groups, sites for vaccination delivery, and recording of information on administered vaccines (Table 2) is based on eleven responding countries. Four countries (Algeria, Bosnia and Herzegovina, Kosovo and Ukraine) did not provide this information.

Three countries (Armenia, Moldova and Palestine) routinely verify the immunization status of all newly arrived migrants. Three countries reported verifying immunization status only among specific population groups: children arriving from countries endemic for tuberculosis or meningitis in Israel, and students coming from certain African countries in Tunisia. Finally, in Georgia, people coming from Nigeria, Syria, Afghanistan and Pakistan are assessed for poliomyelitis immunity.

Immunization status is generally verified through anamnesis and verification of the individual vaccination card. Only Moldova reported using serological testing for hepatitis B, measles, rubella diphtheria and tetanus.

Four countries (Albania, Jordan, Republic of Macedonia-FYROM and Serbia) do not routinely verify the immunization status of newly arrived migrants (Table 2, Section 1).

### 3.3. Target Groups and Vaccinations Offered to Newly Arrived Migrants

Concerning the target age groups for vaccination offers among newly arrived migrants (Table 2, Sections 2 and 3), 8 countries (Albania, Armenia, Egypt, Israel, Jordan, Moldavia, Palestine, Tunisia) reported offering all the vaccinations included in their National Immunization Plan (NIP) to newly arrived migrant children. In Egypt, children below 2 years of age are vaccinated according to the Egyptian national schedule and children under 4 years receive all missed doses according to the national schedule. In addition, an extra dose of bivalent oral poliomyelitis vaccine (bOPV) is offered to children of any age (as well as to adolescents and adults) coming from any country declared to have a polio epidemic according to the latest updated report of the international health emergency committee (according to International Health Regulations 2005) [37]. An extra bOPV dose is also offered to children who have no proof of vaccination in the previous 12 months. In Israel, in addition to routine vaccinations given to all other migrant children, migrants from the horn of Africa are offered meningococcal and Bacillus Calmette-Guerin (BCG) vaccines.

Three countries reported offering the following selected vaccinations to newly arrived migrants: Georgia offers poliomyelitis vaccine to people arriving from Nigeria, Pakistan, Afghanistan and Syria; Republic of Macedonia-FYROM offers poliomyelitis and MMR vaccines; and Serbia offers poliomyelitis, MMR and diphtheria-tetanus-pertussis (DTP) vaccines (Table 3).

Most countries also offer immunization to adolescent and adult migrants (10 and 8 respectively), mainly focusing on MMR, diphtheria-tetanus (dT) and/or poliomyelitis vaccination, as described in Table 3.

### 3.4. Sites for Vaccination Delivery to Newly Arrived Migrants

As shown in Table 2 Section 4, vaccinations are provided at community level in Armenia, Palestine and Serbia. In Albania, Israel, Jordan and Moldavia, migrants mainly receive vaccinations at community level; however, priority vaccines are also offered at holding centre level. Israel has also established pre-entry immunization programmes in public health clinics in Ethiopia targeting Jews travelling to Israel and offering meningococcal vaccine before leaving the country. In Egypt, childhood vaccinations are given at community level, while the extra-dose of bOPV vaccine is administered within the entry site (Table 3).

In the remaining two countries, vaccinations are administered to newly arrived migrants only at entry level. Georgia offers oral poliomyelitis vaccine (OPV) to people arriving from at-risk territories (Nigeria, Syria, Afghanistan and Pakistan) within vaccination service points in airports and seaports. The Republic of Macedonia-FYROM offers MMR and poliomyelitis vaccine at entry level, in two transit centres set at both borders. During 2015, migrants in the Republic of Macedonia-FYROM stayed on average 48 h or less before transiting through the country.

### 3.5. Recording of Data on Vaccines Administered to Newly Arrived Migrants

In most countries (Albania, Armenia, Georgia, Israel, Jordan, Moldova, Palestine), migrants receive an immunization card. Data on the administered vaccines are recorded in a paper and/or electronic registry (Table 2, Section 5). In Georgia, all persons who receive poliomyelitis vaccine at airport/seaport medical service points receive an immunization card. This data are not entered in the vaccination registry for the general population; however, monthly reports on all administered immunizations are sent and collected at the national level. Cases of refusal are promptly reported to the National Center for Disease Control and Public Health.

In Serbia, only an immunization card is provided to migrants. Instead, in Egypt and Republic of Macedonia-FYROM data on any administered vaccines are entered in an immunization registry.

## 4. Discussion

Information on vaccination strategies targeting migrants across the Mediterranean migration system is limited. The Promovax project (2010–2013) recorded existing migrant immunization policies, legislation, and practices in selected EU countries (Germany, Norway, Italy, Poland, Greece, Hungary, Croatia and Cyprus) [38] and analyzed several factors that may influence immunization acceptance in a list of 10 selected migrant ethnicities [39]. However, the project focussed only on regular migrants regardless of the length of stay in the country. In 2015, the European Centre for Disease Prevention and Control (ECDC) collected some information on vaccinations offered to irregular migrants and asylum seekers from EU/EEA countries [40]. An ongoing study of the EU-funded CARE project (“Common Approach for REfugees and other migrants’ health”) [41] is also addressing vaccination strategies; its focus is specifically on six EU countries (Croatia, Greece, Italy, Slovenia, Malta, and Portugal).

To our knowledge, this is the first study performed to explore immunization strategies targeting specifically newly arrived migrants with a focus on non EU-countries of the Mediterranean Area and the Black Sea basin.

We found that, in the majority of the countries we studied, strategies for immunizing newly arrived migrants are in place. Strategies vary largely according to the organization of the health systems, the geographic area and neighbouring countries, migration flows and the pattern of immigration. Notwithstanding, all countries target children who are generally offered all the vaccinations included in the NIP of the hosting country. Although the number of doses and schedules vary among countries, all of them have a NIP and consolidated childhood vaccination programmes against poliomyelitis, measles, rubella, diphtheria, tetanus, pertussis, *Haemophilus influenzae* type b, and hepatitis B. Most of them also offer BCG vaccine. About half of the surveyed countries immunize children against pneumococcus and rotavirus. Israel offers vaccinations also against varicella and hepatitis A [42].

Offering all vaccinations included in the NIP allows them to protect all unimmunized children (and those with unknown immunization status) as soon as possible. Migrants, in fact, often come from countries where civil unrest and wars have damaged health services and interrupted vaccination programmes and might have been unable to initiate or conclude the immunization series.

Some of the surveyed countries have also extended their vaccination offer to adolescent and adult migrants. In this case, as per WHO-UNHCR-UNICEF joint recommendations [12], priority is given to vaccinations against poliomyelitis, measles and rubella, diseases targeted for elimination [43,44]. We found that only half of the surveyed countries have included adults as a target group for migrant immunization and a survey conducted in 2009 [28] found that adult migrants have more limited access to free-of charge services. This finding needs to be put in context by specifying that, in EU [45] and non-EU countries [42], immunization programmes targeting the adult resident population are also generally less consolidated, if compared to childhood immunization programmes.

Recommendations in place in the Mediterranean Area and the Black Sea show some similarities with those existing in EU/EEA countries. Of the 17 EU/EEA countries that replied to the 2015 ECDC request, 12 reported offering children all age-appropriate vaccinations as per their NIP, two countries gave priority to certain vaccines (mainly polio and MMR) and three countries reported not offering vaccinations yet although the item was under discussion at the time of the ECDC request (September 2015) [40]. A lower proportion of responding EU/EEA countries (5/17) reported including also adult migrants in their vaccination offer that mainly included MMR, a diphtheria-tetanus booster and inactivated polio vaccine (IPV) for asylum seekers from polio-risk areas, according to WHO-UNHCR-UNICEF joint guidance [12] and ECDC indications [2,46].

In our survey, we also explored sites for vaccination delivery. While the WHO-UNHCR-UNICEF recommendations state that refugees, asylum seekers and migrants should be immunized without delay, they do not recommend immunization at border crossings unless there is an outbreak of a VPD in the host or transit country [12]. In line with these indications, vaccinations are delivered at community level in most of the countries we studied. Many vaccines require two or three doses at timed intervals and the follow up of the full immunization series. Starting/updating vaccinations after the integration of migrants, through the same health services used by the local population, might facilitate the administration of full vaccination cycles by planning and respecting scheduled time intervals. On the other side, barriers in accessing community health services might unnecessarily delay the administration of first doses, especially while people are on the move.

This finding is in line with the 2009 survey mentioned above [28] that found that the predominant vaccination delivery pattern for migrants was through community-based services also targeting the general population in EU and non EU countries of the Mediterranean basin. The authors commented that, although in principle migrant populations were reported as eligible for these services, informal barriers (linguistic, cultural, etc.) could be fostered due to the lack of migrant sensitive approaches to service delivery.

In our survey, only a minority of countries provide vaccinations at the entry level, confirming a mainly community-based service delivery model: in Georgia polio vaccine is given to people coming from high-risk countries and in Republic of Macedonia-FYROM only poliomyelitis and MMR vaccines are provided. Egypt delivers all childhood vaccinations at the community level, while the extra-dose of polio vaccine is offered at entry point to migrants coming from countries declared at risk for poliomyelitis. In this case, the migrants' entrance in the country is used to promptly provide one shot of the priority vaccines as soon as possible, in order not to lose them after their entry in the country.

The WHO-UNHCR-UNICEF guidance also suggests providing documentation of each administered vaccination to the concerned migrant or child’s caregiver to help avoid unnecessary re-vaccinations [12]. In most of the countries that replied to our questionnaire, an individual vaccination card is delivered to migrants and data are entered in electronic or paper immunization registries. However, individual cards are possibly lost during the long journey or destroyed on purpose to eliminate any document that could allow the migrant’s identification for the fear of legal consequences. Furthermore, countries where information on administered doses is archived in national databases might not routinely share their data with other countries. Procedures to keep track of migrants’ immunization data across countries should be improved to avoid lack or duplication of vaccination. This is a challenge as it entails a very strict coordination and collaboration among public health authorities of different countries to define a common format for data sharing and sophisticated infrastructures to exchange electronic data while ensuring confidentiality.

Some pilot projects are ongoing to track the health status of migrants and refugees in order to facilitate follow up and continuity of care. The International Organization for Migration, with the support from the European Commission, is developing and piloting an online health platform (E-PHR, Electronic Personal Health Record) to archive migrants’ health information and to ensure that migrant health assessment records are available, under strict data protection rules, at transit and destination countries [47,48]. Concomitantly, a paper format of the Personal Health Record (PHR), was produced. This document is designed to be compiled by healthcare workers after clinical consultations and left with the consulted migrant/refugee who can then show it to the healthcare workers he/she may subsequently encounter during the journey [47]. Building upon the PHR, the CARE project is developing an integrated electronic system that encompasses a portable device to be delivered to migrants and refugees, containing their personal medical history, as well as information on any treatment provided [49].

Our survey has certain limitations. As the questionnaire was filled by an expert at the national level, we collected information on national immunization strategies targeting newly arrived migrants but could not investigate how those strategies are implemented at the local level. Moreover, as this study was conducted as part of a wider survey, it was not possible to introduce more detailed questions in order not to overload the countries’ experts in charge of filling the questionnaire, thus improving the participation rate. For this reason, we could not investigate if variability at sub-national level exists and how vaccinations are accepted by migrants. Access of migrants to centre/community health services, vaccination coverage and proportion of completed cycles were also not assessed.

Another limitation is that we used a broad definition of “migrant”, that includes diverse population groups (such as refugees and asylum seekers, undocumented migrants, economic migrants, students) with different legal status and, consequently, possibly different access to vaccination. We were therefore unable to explore how immunization offers vary across these different groups.

Finally, given the short format of the survey, a higher number of piloting countries would have reduced the risk of bias in the interpretation of the questions.

## 5. Conclusions

This study provides a first overview of current immunization strategies targeting newly arrived migrants in non EU-countries of the Mediterranean Area and the Black Sea basin. Although diversified, implementation strategies for immunizing newly arrived migrants are in place in most of the surveyed countries, mainly targeting unvaccinated children or those with an unknown immunization status.

Migrants are considered a vulnerable group for certain infectious diseases because they can originate from disease-endemic countries and because they can be exposed to poor living conditions and overcrowding during their arduous journeys. Their desired destinations are countries with decreasing immunization coverage levels and pockets of susceptible populations. As migratory pressure is increasing population movements across borders, all countries should play their role in identifying unprotected people (or those with unknown immunization status) and ensure the implementation of appropriate immunization programmes for refugees, asylum seekers and migrants [50]. Vaccination, in fact, represents a tool for the greater benefit of all, protecting both the health of migrants and of the host community. For this reason, immunization offers to migrants should be regulated and guaranteed in all countries and acceptance should be monitored.

Our study highlighted a widespread immunization offer to migrant children according to the NIPs. However, from our results, we cannot infer how existing strategies are implemented at the local level, whether they are currently functioning and whether, and what, barriers to their implementation are being faced. Further studies focusing on these aspects are needed to develop concrete and targeted recommendations for action. In addition, a more focused study aiming to assess immunization entitlement gaps among less targeted population groups, such as adults and/or more disadvantaged migratory status groups, such as irregular migrants, is needed.

At the same time, more efforts such as development of online immunization registries and the promotion of cooperation between countries of origin, transit and destination, should be encouraged in order to share immunization strategies and monitor administered vaccines. The finalization, sharing and dissemination of the pilot projects mentioned above [47,48,49] could represent useful steps to track the health status of migrants and refugees, establishing mechanisms of collaboration among countries. This could guarantee appropriate and targeted health assistance for migrants/refugees and facilitate cross-border immunization planning. Furthermore, effective health status monitoring can also lead to avoiding unnecessary health actions, including unnecessary re-vaccination.

## Figures and Tables

**Table 1:** Availability of specific regulations/procedures supporting immunization of newly arrived migrants in countries from Mediterranean Area and Black Sea Basin (N = 15).

Availability of Regulations/Procedures for Immunization of Newly Arrived Migrants	Number of Countries (n/N)	% ^1^
No regulations/procedures	6/15	40
Regulations/procedures at the national level	4/15	27
Regulations/procedures in some geographical areas	1/15	7
Regulations/procedures for certain population groups	4/15	27
Other ^2^	1/15	7

^1^ The sum of percentage exceeds 100% because one country (Israel) reported regulations/procedures at the national level as well as provisions for specific population groups. ^2^ Kosovo stated that according to the plan prepared by Ministry of Health in case of a migration influx, immunization priority would be given to the vaccination of children aged less than 5 years against measles and poliomyelitis.

**Table 2:** Verification of immunization status of newly arrived migrants, vaccinations offered, target groups, sites of vaccination delivery and registration of data on administered vaccines in countries from Mediterranean Area and Black Sea Basin (N = 11).

Characteristics of Vaccination Offer to Newly Arrived Migrants	Number of Countries (n/N) ^1^	%
**1. Verification of migrants‘ immunization status**		
Immunization status routinely verified	3/10	30
Immunization status verified only among specific population groups	3/10	30
Immunization status not routinely verified	4/10	40
**2. Target age groups for vaccination offer to newly arrived migrants**		
Only children	1/11	9
Children and adolescents	2/11	18
Children, adolescents and adults	8/11	73
**3. Childhood vaccinations offered to migrants**		
All the vaccinations included in the National Immunization Plan	8/11	73
Only certain vaccinations are offered to migrants	3/11	27
**4. Sites for vaccination delivery**		
Entry level	2/10	20
Entry and community level	1/10	10
Holding centres and community level	4/10	40
Community level	3/10	30
**5. Recording of data on administered vaccines**		
Individual immunization card is given to migrants and data are recorded in a registry	7/10	70
An individual immunization card is given to migrants	1/10	10
Data are registered in a paper and/or electronic registry	2/10	20

^1^ Information on verification of immunization status, sites for vaccination delivery and registration of data on administered vaccines was provided by 10/11 countries.

**Table 3:** Vaccinations offered to newly arrived migrants by age group (children, adolescents and adults) and site for vaccination delivery, in countries from Mediterranean Area and Black Sea Basin.

Country	Children	Adolescent	Adult	Site for Vaccine Delivery
Albania	All vaccinations according to the NIP ^1^	dT, poliomyelitis, MMR	dT, poliomyelitis, MMR	Detention centres; community level
Armenia	All vaccinations according to the NIP	dT, MMR, poliomyelitis	dT, MMR	Community level
Egypt	All vaccinations according to the NIP for children less than 4 years Poliomyelitis vaccine to children at any age coming from a country at polio risk	Poliomyelitis vaccine to arrivals from a country at polio risk	Poliomyelitis vaccine to arrivals from a country at polio risk	Community level; Poliomyelitis vaccination at entry level
Georgia	Poliomyelitis vaccine to arrivals from Nigeria, Syria, Afghanistan and Pakistan	Poliomyelitis vaccine to arrivals from Nigeria, Syria, Afghanistan and Pakistan	Poliomyelitis vaccine to arrivals from Nigeria, Syria, Afghanistan and Pakistan	Entry level in airports and seaports
Israel	All vaccinations according to the NIP; Meningococcal and BCG vaccine to arrivals from Horn of Africa (BCG to children <4 years)	All vaccinations according to the NIP to adolescents aged 11–14 years; Poliomyelitis vaccine to adolescents aged 11–17 years; Meningococcal vaccine to arrivals from Horn of Africa	Meningococcal vaccine to arrivals from Horn of Africa; vaccination in case of outbreaks	Community level; Meningococcal vaccine for Ethiopian Jews at public health clinics set in Ethiopia by Israeli government
Jordan	All vaccinations according to the NIP, specially for Syrian refugees	Measles to adolescents aged 11–15 years	Tetanus to child bearing age females (15–49 years) according to NIP	Community level; Measles vaccine to people 6 months–15 years and poliomyelitis vaccine to children <5 years also in holding centres
Moldova	All vaccinations according to the NIP	All vaccinations according to the NIP	All vaccinations according to the NIP (hepatitis B vaccine to risk groups)	Community level; Poliomyelitis, DTP, MR, pneumococcal vaccine also in holding centres
Palestine	All vaccinations according to the NIP			Community level
Republic of Macedonia-FYROM	Poliomyelitis and MMR vaccine	Poliomyelitis and MMR vaccine		Entry level in two transit centres
Serbia	Poliomyelitis, DTP, MMR vaccine	Poliomyelitis, DTP, MMR vaccine	Tetanus according to NIP	Community level
Tunisia	All vaccinations according to the NIP, with particular attention to Libyan and Syrian foreigners	Poliomyelitis, DTP, MMR vaccine		

^1^ NIP: National Immunization Plan.
